# Motivational Mindsets and Reasons for Studying: Development and Validation of a Classification Tool

**DOI:** 10.3389/fpsyg.2020.535801

**Published:** 2020-12-17

**Authors:** Job Hudig, Ad W. A. Scheepers, Michaéla C. Schippers, Guus Smeets

**Affiliations:** ^1^Erasmus School of Social and Behavioural Sciences, Erasmus University, Rotterdam, Netherlands; ^2^Rotterdam School of Management, Erasmus University, Rotterdam, Netherlands

**Keywords:** mindset, motivation, student, person-centered, cluster analysis

## Abstract

First-year university students have multiple motives for studying and these motives may interact. Yet, past research has primarily focused on a variable-centered, dimensional approach missing out on the possibility to study the joint effect of multiple motives that students may have. Examining the interplay between motives is key to (a) better explain student differences in study success and wellbeing, and (b) to understand different effects that interventions can have in terms of wellbeing and study success. We therefore applied a student-centered, multidimensional approach in which we explored motivational profiles of first-year university students by combining three dimensions of motives for studying (self-transcendent, self-oriented, and extrinsic) which have been shown to be differently related to academic functioning. Using cluster analysis in two independent, consecutive university student cohorts (*n* = 763 and *n* = 815), we identified four meaningful profiles and coined them motivational mindsets. We validated the four mindset profiles not only within each student sample but also found almost identical profiles between the student samples. The motivational mindset profiles were labeled: high-impact mindset, low-impact mindset, social-impact mindset, and self-impact mindset. In addition to validating the paradigm, we developed a mindset classification tool to further use these mindsets in practice and in future research.

## Introduction

Imagine Oliver, he is a first-year university student. He just turned 18 and in high school, he passed his exams with flying colors without having to put in much effort. Oliver chose to go to university because he wants to get a degree, but moreover it is a priority for him to have an active social life during his studies. He picked his study program mainly because of his friends and together they compete to obtain the highest grades. Then imagine Aisha, she is an 18-year-old first-year university student and she chose to go to university because she prepares for her future career. She aims to develop relevant skills and knowledge and the curriculum of the study program attracted her to reach this goal. Aisha wishes to earn her degree with honors to increase her opportunities at the job market when she finishes her studies.

These examples show that students come to university with a certain mindset (i.e., a frame of mind) that relates to their motives for studying. The examples also display that this mindset can consist of multiple motives simultaneously. So far, research on motives for studying has primarily been dimensional or variable-centered (Yeager et al., 2014). This traditional method tests the relationship between one dimension of study motives and one or more outcomes but lacks the ability to elicit the joint effect of multiple motives (Vansteenkiste et al., 2009). As students have multiple motives and these motives may interact, a student-centered, *multidimensional* perspective on motives for studying can be more fruitful to (a) explain student differences in first-year study success and wellbeing, and (b) understand why interventions are effective for some first-year students and not for others.

Past research has investigated what students’ motives for studying represent and where they emanate from (e.g., Henderson-King and Smith, 2006; Kember et al., 2008; Eccles, 2009; Stephens et al., 2012; Kennett et al., 2013; Twenge and Donnelly, 2016). A specific body of research has identified several dimensions of motives which showed to be important for academic functioning and psychological wellbeing (Yeager and Bundick, 2009; Yeager et al., 2012, 2014). Yet, these dimensions have not been studied other than in high-school settings. Moreover, in terms of study motives, the dimensions have only been researched following a variable-oriented approach and the interplay between them still has to be examined. To fill this gap, we explored whether we could construct *motivational mindsets* on the basis of first-year university students’ motives for studying. Mindsets are frames of mind that orient individuals toward corresponding actions and responses (Crum et al., 2013). Up until now, mindsets have predominantly been studied as individual beliefs regarding the malleability of a particular skill or ability (Dweck, 1999). Consequently, the categories within this particular type of mindset have shown to be influential for resilience (Yeager and Dweck, 2012), creativity (Redifer et al., 2019), stress (Crum et al., 2017), wellbeing (Van Tongeren and Burnette, 2016), and academic achievement (Costa and Faria, 2018). In the current paper, we coin the term motivational mindsets to refer to the combination of motives for studying that predispose students to reactions on their studies and study environment.

The approach was to examine how individual students differ in their unique combinations of motives and to identify homogeneous subgroups of students (better known as *profiles*) that share similar patterns. This *person-centered approach* provides a novel viewpoint by concentrating on individual profiles of students rather than specific relations among variables (Vansteenkiste et al., 2009). The current investigation with large cohorts of first-year students aimed to extend the knowledge about how these students differ in their motivation for learning when they start their studies. The academic relevance entails that a multidimensional perspective of study motives was adopted to better and more fully understand first-year student motivation. As far as the societal relevance, gaining new types of motivational mindset profiles would be instructive to investigate how and why educational interventions affect groups of students differently.

### Theoretical Background

Little research has been conducted in education on motivational profiles using the person-centered approach (Gillet et al., 2017). Prior studies fusing student motivation with student-centered analyses have focused on the expectancy-value framework (Watt et al., 2019); achievement goal-orientation (Tuominen-Soini et al., 2011, 2012); need achievement (Covington and Omelich, 1991); burnout and engagement (Salmela-Aro and Read, 2017); and combinations of adaptive/maladaptive motivation, self-regulation, and psychological wellbeing (Elphinstone and Tinker, 2017). Among the research that has focused on the multidimensionality of motivation, the most prominent in the academic context has primarily been carried out from the self-determination framework (Deci and Ryan, 2000).

Self-determination theory (SDT) has a multidimensional perspective of motivation and distinguishes several dimensions that are important to explain how students regulate their behavior. Many of the SDT studies incorporating student profiles either combined extrinsic and intrinsic motivation (e.g., Hayenga and Corpus, 2010) or autonomous and controlled motivation (e.g., Vansteenkiste et al., 2009). Intrinsic motivation pertains to engaging in a task for its own inherent rewards whereas extrinsic motivation pertains to engaging in a task in order to obtain some separable outcome (Hayenga and Corpus, 2010). Autonomous motivation refers to students feeling that they are the agents of their own behavior (Mouratidis and Michou, 2011). This dimension of motivation is fueled by three types of motivation: (a) the inherent enjoyment that students experience in their studies (intrinsic motivation), (b) whether students view the study activities as personally important (identified regulation), and (c) whether students fully assimilate studying into their sense of self (integrated regulation). In contrast, controlled motivation refers to students regulating their behavior based on external factors that are internalized (Mouratidis and Michou, 2011). This dimension has two distinct types of motivation: (a) performing study-related actions to avoid feelings of guilt and shame or to sustain self-worth (introjected regulation), and (b) executing study activities to obtain rewards or avoid punishments (external regulation). Lastly, SDT includes the possibility that students possess neither autonomous nor controlled motivation which is called amotivation. The framework follows a continuum from intrinsic motivation to amotivation and, more importantly, the types of motivation in SDT are not mutually exclusive. Despite the value of SDT in student-centered research, the emphasis in this motivational framework has particularly been from a self-interest perspective. We decided therefore to not focus on autonomous and controlled motivation, nor solely on intrinsic and extrinsic motivation, but also to include the self-other dimension.

Following earlier research, the current study framed the underlying reasons that motivate first-year students to study in three distinct dimensions of motives that vary to the extent that they are intrinsic self-oriented, intrinsic self-transcendent, and extrinsic self-oriented (Yeager et al., 2014). *Intrinsic self-oriented motives* refer to reasons for studying because it is inherently interesting or enjoyable. These motives encompass students’ aspirations to benefit themselves, whereby going to university to improve their skills and knowledge would make their lives more gratifying or personally meaningful (Yeager et al., 2014). *Intrinsic self-transcendent motives* refer to reasons for studying because it provides students opportunities to contribute to a better world. These motives transcend self-interest in which their studying has the potential to have some effect on or connection to the world beyond the self (Yeager et al., 2014). *Extrinsic self-oriented motives* refer to the performance of studying in order to attain some separable outcome (e.g., money or status). Even if this money would be used to make the world a better place, the student would still aspire for a separable outcome and thus the motive is extrinsic in nature (Yeager et al., 2012, 2014).

Yeager et al. (2012) conducted interviews with middle- and high-school students regarding their career goals after which they applied a person-centered approach by categorizing students into four groups: no intrinsic motives, only intrinsic self-oriented motives, only intrinsic self-transcendent motives, and both intrinsic motives. This categorization was carried out based on what type of reasons students mentioned during the interview. The results showed that only students who mentioned both intrinsic self-oriented motives and intrinsic self-transcendent motives for their future career were more likely to have a higher sense of purpose and were also more likely to find their schoolwork highly meaningful. Yeager et al. (2014) subsequently conducted a quantitative study with students in their final semester of high school. Besides the intrinsic study motives, they now included extrinsic self-oriented motives as well and created a new measurement instrument focusing on these three dimensions of students’ motives for studying. The findings showed that having self-transcendent motives for studying predicted greater task persistence and personal meaningfulness of schoolwork. The intrinsic self-oriented motives and the extrinsic self-oriented motives, on the contrary, did not predict greater self-regulation nor meaningfulness of schoolwork. In fact, the extrinsic motives showed to be negative predictors of both personal meaningfulness of schoolwork and academic self-regulation. Because students’ reasons for going to university have shown to be differently related to academic functioning and psychological wellbeing, but also given that self- and other-focused processes intertwine (Crone and Fuligni, 2020), we wanted to further investigate the interplay between these three types of motives within students.

### Present Study

The purpose of the present study was to develop motivational mindset profiles of students on the basis of their levels of intrinsic motives (hereafter: self-oriented motives and self-transcendent motives) and extrinsic motives for studying. This study is to the best of our knowledge the first study that employed these three dimensions of study motives to generate individual profiles of students. We aimed to answer the following central research question: “Can meaningful motivational mindset profiles, combining the three dimensions of study motives, be identified among first-year university students?” In this paper, two independent samples of first-year university students were used to investigate the multidimensionality of the study motives. First, we provide the sample data and examine whether the samples have similar composition and characteristics. Subsequently, we explore the research question through cluster analysis and then test the quality of the cluster solutions in each sample separately. After we identified the best set of meaningful profiles, we describe and label them accordingly as mindsets. Finally, we extend and enrich the characterization of the mindsets in the discussion and indicate promising avenues for future research.

## Materials and Methods

### Sample and Procedure

We included two independent samples of first-year students from two consecutive cohorts (academic years 2017–2018 and 2018–2019) in a Dutch business administration bachelor’s degree program. The dataset including both samples was permanently anonymized by an authorized person of the research group and, since the results were impossible to link back to the original data of the respondents, the research was compliant with the General Data Protection Regulation (GDPR). To anonymize the dataset and to check whether the samples had similar composition in age, we categorized the samples into three age groups: under 18, 18–20, and 21 and older. As we replicated the person-centered analysis in the second sample, we also examined whether the samples had similar composition in terms of gender and ethnicity characteristics. Sample 1 consisted of 778 students and sample 2 consisted of 852 students. However, after screening the data (see section “Preliminary Analysis”), the final sample size was 763 students in sample 1 and 815 students in sample 2. In sample 1, 6.8% of the students were under 18, 88.4% were 18 to 20, and 4.8% of the students were 21 and older. Furthermore, 67.0% were male and 18.1% represented non-Western ethnic minority students. In sample 2, 9.0% of the students were under 18, 86% were 18 to 20, and 4.7% of the students were 21 and older. Additionally, 67.2% of sample 2 were male and 14.1% represented non-Western ethnic minority students. From this it shows that the vast majority of both samples fall in the 18 to 20 age group. Also, the composition of the samples corresponded well with each other on the basis of gender and ethnicity data. The data were collected 3 weeks after the start of the program. A questionnaire including items about mood, personality, motivation and other psychological constructs was administered before the start of an intervention which was part of an introductory course on Managerial Skills. Tutors explained the purpose of the intervention and the questionnaires during the regular group meetings and they asked students to participate in the research. They informed students that participation in the questionnaires was voluntary and not participating had no consequence for their grades. The questionnaire was then distributed by Qualtrics software and students were given 1 week to complete the questionnaire via this platform. Data on gender and age and ethnicity were gathered from the academic transcripts of the university and all methods concerning the intervention study were carried out in approval of the research school’s Internal Review Board.

### Measures

Study motives were assessed with an adapted version of the 10-item instrument designed by Yeager et al. (2014). The items and the reliability of the subscales from the original study are described below. The research group transformed this original *motives for going to college* measure into the study motives measure by adapting it to the university context and translating it into Dutch. When the measure was initially adopted and used by the research group, we did not use a translation-back-translation method. Although this limitation was hard to overcome after the data was already collected, an extra procedure was conducted to verify the linguistic quality of the measure. Three proficient English users independently translated the Dutch version back into English. From their assessments, it showed that the back-translations corresponded almost perfectly with the original version and the meaning of the items was considered equivalent. Because of similar samples (i.e., high school students in their final semester and university students in their first semester), the meaning of the preface “How true for you personally are each of the following reasons for going to college?” was altered to the statement “I chose to go to university because” and then the ten items followed. Similarly, all items were rated on a scale ranging from 1 to 5; however, different response options (I totally disagree to I totally agree) were applied to equal the other measures in the questionnaire to not confuse the participants and to limit the length of the questionnaire.

Self-transcendent motives consisted of the following three items: “I want to learn things that will help me make a positive impact on the world,” “I want to gain skills that I can use in a job that help others,” and “I want to become an educated citizen that can contribute to society.”

Self-oriented motives consisted of the following three items: “I want to expand my knowledge of the world,” “I want to become an independent thinker,” and “I want to learn more about my interests.”

Extrinsic motives consisted of the following four items: “I want to get a good job,” “I want to leave my parents’ house,” “I want to earn more money,” and “I want to have fun and make new friends.” These items of the original scale were created in collaboration with counselors at the participating schools.

### Analyses

The quality of our study motives measure was assessed in the two samples through exploratory factor analysis, composite reliability scores and Cronbach alpha values for each study motives subscale. To answer our research question, *k*-means cluster analysis was performed in each sample separately. All analyses were executed using SPSS version 25. The *k*-means algorithm tracked naturally occurring score patterns in the three dimensions of study motives and then grouped individual responses into profiles based on similar patterns (Daniels et al., 2008). The goals in *k*-means cluster analysis are to detect between-cluster heterogeneity and within-cluster homogeneity (Chittum et al., 2019). Neuville et al. (2007) have argued that this iterative method is appropriate for large datasets (>150 subjects) and because the method makes more than one pass through the data instead of one (as with hierarchical methods) it can compensate for low initial partition of the data. Notably, clustering techniques such as *k*-means have been regarded as less optimal than model-based techniques because they are more subjective (e.g., Meyer and Morin, 2016). However, a large simulation study has shown that *k*-means can perform as well as model-based approaches to identify underlying profiles (Steinley and Brusco, 2011). Moreover, a meta-analytic investigation of empirical, person-centered studies has supported this conclusion as highly similar results were identified between model-based and non-model-based techniques (Wormington and Linnenbrink-Garcia, 2017).

Considering the inclusion of three profiles variables and in line with several previous person-centered studies on student motivation (e.g., Vansteenkiste et al., 2009; Hayenga and Corpus, 2010; Wormington et al., 2012; Chittum and Jones, 2017; Robinson et al., 2017; Chittum et al., 2019), we expected the *three*-, *four*-, *and five*-cluster solutions as potentially workable. The criteria for the best cluster solution consisted of theory, cluster sizes, parsimony, and explanatory power (Vansteenkiste et al., 2009). Multivariate analysis of variance was used to discern the potential explanatory power and to examine the variability in the cluster solutions. Subsequent univariate main effects were considered and had to confirm at least 50% of the variance in each of the dimensions of study motives (Breckenridge, 2000; Vansteenkiste et al., 2009). Following guidelines from earlier research, double-split cross-validation procedures were performed in each sample to test the internal validity and stability of the final cluster solution (Breckenridge, 2000). Since we examined two independent student samples, reliability of the cluster solution could be substantiated accordingly. Lastly, cross-tabulation analyses were executed to find differences in distribution of clusters according to gender and ethnicity.

## Results

### Preliminary Analysis

On the item-level, sample 1 included 18 missing values (0.003%) and sample 2 included 15 missing values (0.002%). As the extent of missing values was very small and there did not seem to be any patterns, we decided to impute the series mean to handle them (Tabachnick and Fidell, 2012).

Prior to the factor analysis, we screened both samples for univariate and multivariate outliers and tested recommended assumptions (Hair et al., 2013). In sample 1, five cases had high standardized values and two cases had high Mahalanobis distance values. In sample 2, 12 cases showed high standardized scores on at least one of the variables and four cases had high Mahalanobis distance values. After confirming adequate Cook’s distance values (<1) and, more importantly, observing valid item responses and no problematic patterns, we decided to keep these cases in our datasets.

Subsequently, we conducted univariate and multivariate normality tests. The skewness of the three variables was between −0.25 and −0.73 in sample 1. The variables in sample 2 showed skewness levels between −0.56 and −1.00. A negative skew value implies that the tail on the left side of the distribution is longer than the right side and the majority of the values lie to the right of the mean. An absolute value larger than 2 can be regarded as a substantial deviation from normality (Kim, 2013). The other univariate normality test entails kurtosis which is a measure of the peakedness of a distribution. Here an absolute kurtosis value of 7 can be proposed as a departure from normality (Kim, 2013). In sample 1, the kurtosis values ranged from 0.41 to 2.03 and in sample 2, kurtosis values ranged from 1.07 to 2.62. Despite moderate skewness and kurtosis, values were deemed acceptable. Multivariate normality was then checked via quantile–quantile (Q–Q) plots which displayed that the three distributions deviated from a perfect diagonal line. When the data violate the assumption of multivariate normality, Principal Axis Factor (PAF) is recommended (Costello and Osborne, 2005). Furthermore, linearity was assessed and, as no curves were visible in the data, this assumption was met. Lastly, we adhered to the recommended sample size for exploratory factor analysis of at least 300 participants (Gie Yong and Pearce, 2013).

A principal axis factor analysis was conducted in each sample on the 10 items with direct oblimin rotation. The Kaiser–Meyer–Olkin (KMO) measure confirmed the sampling adequacy for the analysis in both samples, KMO sample 1 = 0.73 and KMO sample 2 = 0.74. In addition, all individual items were above 0.63 in sample 1 and 0.66 in sample 2 which indicated values well above the threshold of 0.5 (Field, 2013). An initial analysis in sample 1 provided four factors with eigenvalues over Kaiser’s criterion of 1, while the scree plot clearly showed a break at three factors. Following the inflection of the scree plot, we decided to retain three factors. When we inspected the factor loadings after rotation, item 8 displayed a low-loading of 0.24. Tabachnick and Fidell (2012) proposed an acceptable limit of factor loadings of 0.32; hence, we considered item 8 as a problematic item. When further inspecting the meaning of item 8 (i.e., I want to leave my parents’ house), its perspective did not resemble the population. The item was originally used for high-school students living with their parents, while students in our population had started their studies assuming that many had already moved from their parents’ house. Based on this theoretical perspective and on the initial results of the factor analysis, we wanted to enhance the quality of the study motives instrument and, therefore, decided to alter the extrinsic motives scale by removing item 8.

Consequently, we transformed the extrinsic motives variable and rerun our entire analysis procedures. Data screening of the new variable did not render reasons for removing cases. Skewness of the extrinsic motives variable now showed values of −0.86 in sample 1 and −1.2 in sample 2. Kurtosis values extended to 2.03 in sample 1 and 3.16 in sample 2. Although the distribution deviated reasonably from normality, both these tests still demonstrated acceptable values (Kim, 2013). A Q–Q plot of the extrinsic motives variable displayed a deviation from a perfect diagonal line which made PAF desirable. Finally, assessment of the plots revealed linear relationships between the variables.

A principal axis factor analysis was conducted in each sample on the 9 items with direct oblimin rotation. The KMO measure confirmed the sampling adequacy for the analysis with KMO = 0.74 in both samples. Additionally, all individual items were above 0.62 in sample 1 and 0.63 in sample 2. The inflections in the scree plots justified retaining 3 factors and Table 1 displays the factor loadings after rotation in each sample.

**TABLE 1:** Standardized factor loadings from the EFA.

Item	ST		SO		EX	
	Sample 1	Sample 2	Sample 1	Sample 2	Sample 1	Sample 2
ST1	**0.59**	**0.59**	–0.09	0.19	–0.09	–0.11
ST2	**0.70**	**0.85**	0.09	–0.11	–0.01	0.01
ST3	**0.45**	**0.32**	–0.13	0.20	0.24	0.27
SO4	0.25	0.06	−**0.42**	**0.76**	0.07	–0.09
SO5	0.08	–0.09	−**0.81**	**0.69**	–0.06	0.03
SO6	0.04	0.05	−**0.49**	**0.48**	0.06	0.09
EX7	–0.07	–0.01	−0.06.	–0.04	**0.72**	**0.74**
EX9	–0.03	–0.14	0.12	–0.01	**0.72**	**0.75**
EX10	0.08	0.13	–0.10	0.09	**0.37**	**0.35**

*The abbreviations refer to the following study motives dimensions: ST = self-transcendent motives, SO = self-oriented motives, and EX = extrinsic motives. Factor loadings over 0.32 appear in bold.*

The self-transcendent motives (α = 0.63 in sample 1 and α = 0.65 sample 2), self-oriented motives (α = 0.63 sample 1 and α = 0.68 sample 2), and extrinsic motives subscales (α = 0.60 in sample 1 and α = 0.62 in sample 2) all had inter-consistency coefficients between 0.6 and 0.7 which is acceptable (Hair et al., 2013; Taber, 2017). When comparing these assessment results to the original research of Yeager et al. (2014), the self-transcendent motives (α = 0.75) and self-oriented motives (α = 0.70) subscales indicated relatively higher coefficients in their study. On the contrary, their extrinsic motives subscale (α = 0.50) showed a fairly lower coefficient than in our investigation. To extend the validity assessment of our study motives instrument, we calculated the composite reliability (CR) values. If the CR value is 0.6 or higher the scale has a reasonable internal consistency (Aubert et al., 1996; Lawson-Body and Limayem, 2004). The self-transcendent motives subscale demonstrated CR values of 0.61 in sample 1 and 0.63 in sample 2. Similarly, the self-oriented motives subscale proved CR values of 0.60 in sample 1 and 0.69 in sample 2. Finally, the extrinsic motives subscale revealed CR values of 0.64 in sample 1 and 0.66 in sample 2. Based on these calculations, adequate construct validity could be confirmed. Considering the sample reliability, two academic cohorts of a business study program were studied. Apart from the homogeneous character, these cohorts could be included completely and thus the population of first-year business students was well represented. Nonetheless, the reliability coefficients were low by most standards (<0.8). Moreover, as we used questionnaires, these were likely to contain measurement errors. To date, no methods are available to correct clustering techniques for such uncertainty in the data.

As cluster analyses are extremely sensitive to outliers (Tabachnick and Fidell, 2012), we calculated *Z*-scores and Mahalanobis distance values to check for univariate and multivariate outliers. The cut-off criterion was set at a mean distance of three standard deviation units. As a result, 15 univariate outliers were removed in sample 1 (i.e., 1.90% of the sample) and 37 univariate outliers in sample 2 (i.e., 4.34% of the sample). Subsequently, Mahalanobis distance was computed to identify multivariate outliers. No additional cases were excluded from analysis based on these values, which yielded a final sample size of 763 cases in sample 1 and 815 cases in sample 2. Finally, normality was checked. As skewness and kurtosis were between 0 and 1 for all composite variables, acceptable ranges of normality were established.

Table 2 displays the descriptive statistics for the measured variables and intercorrelation coefficients of the three variables. The descriptives show that the means and standard deviations were nearly equal across the two samples. The correlation coefficients show medium correlation (*r* = 0.39 in both samples) between the self-transcendent motives and self-oriented motives variables (Cohen, 1988; 0.10 low, 0.30 is medium, 0.50 is high). The extrinsic motives variable correlated lowly with the self-transcendent motives variable (*r* = 0.15/0.12) as well as with the self-oriented motives variable (*r* = 0.15 in sample 1 and *r* = 0.17 sample 2). All these coefficients were statistically significant and given that the highest correlation coefficient was 0.39, multicollinearity was not a concern (Hair et al., 2013).

**TABLE 2:** Descriptive statistics, Cronbach’s alpha coefficients, and intercorrelations.

		*M*		*SD*		α				*r*			
	Number of items							1		2		3	
		Sample 1	Sample 2	Sample 1	Sample 2	Sample 1	Sample 2	Sample 1	Sample 2	Sample 1	Sample 2	Sample 1	Sample 2
(1) Self-transcendent motives	3	3.95	3.99	0.52	0.53	0.63	0.65	–					
(2) Self-oriented motives	3	4.15	4.20	0.53	0.53	0.63	0.68	0.39**	0.39**	–			
(3) Extrinsic motives	3	4.25	4.31	0.53	0.52	0.60	0.62	0.15**	0.12^∗∗^	0.17**	0.21**	–	

n = 763 in sample 1 and n = 815 in sample 2. ** p < 0.01, All correlations are significant < 0.01.

### Cluster Analysis

**FIGURE 1:**
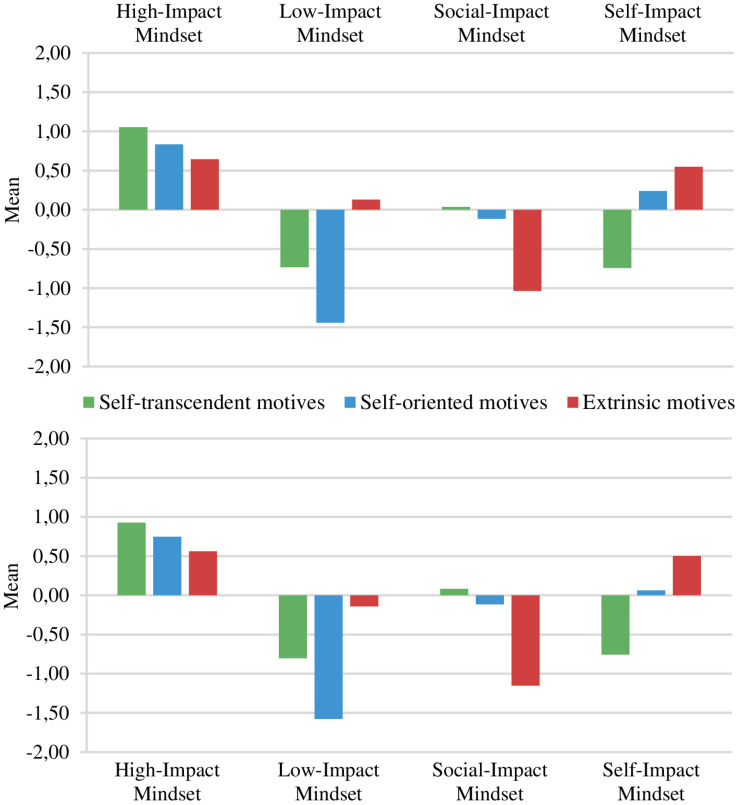
**(Top)** *Z*-scores of self-transcendent motives, self-oriented motives, and extrinsic motives of the four-cluster solution, Sample 1 (*n* = 763). **(Bottom)** *Z*-scores of self-transcendent motives, self-oriented motives, and extrinsic motives of the four-cluster solution, Sample 2 (*n* = 815).

We conducted the exact same analyses on each sample consecutively, starting with sample 1. For the purpose of this study, we report the results simultaneously in the current section. *Z*-scores of the study motives dimensions were employed to cluster the students into different motivational mindset profiles. *k-*means cluster analyses were executed in accordance with *k*-means cluster procedures and provided cluster centers (i.e., means) for each study motive dimension in a *three*-, *four*-, and *five-*cluster solution. Importantly, as the study motives variables were standardized, the cluster center scores do not represent a high score by itself but a value *relative* to the overall sample mean. We examined the variance explained, cluster sizes, and theoretical interpretability/consistency for the profiling variables (self-transcendent motives, self-oriented motives, and extrinsic motives) in each of cluster-solutions. As shown in Table 3, the three-cluster solution explained an insufficient amount (<50%) of variance in two out of three profiling variables (Vansteenkiste et al., 2009). The amount of explained variance in the four- and five-cluster solutions were above the threshold of 50% and showed to be fairly similar. Although the five-cluster solution explained a slightly higher amount of variance in each profiling variable, the meaning of the five-cluster solution did not differ from the four-cluster solution. Both in sample 1 and sample 2, the five-cluster solution produced similar profiles as in the four-cluster solution, yet with one theoretically uninterpretable profile. Moreover, the four-cluster solution emerged as almost identical between the two samples which provided an initial sign of stability. Additionally, the clusters in the four-cluster solution comprised a well-balanced number of students (around 15–35% of the sample). Therefore, on the basis of explanatory power, theoretical interpretability, parsimony, and cluster sample sizes, we chose the four-cluster solution as the best set of profiles. Table 4 and Figure 1 display the four clusters obtained with final cluster centroids in sample 1 and sample 2. A one-way MANOVA was computed with cluster membership as the between subjects factor and the three study motives dimensions as dependent variables. The overall MANOVA was significant in both samples and Roy’s largest root was 2.08, *F*(3,762) = 525.86, *p* < 0.001 in sample 1 and 2.14, *F*(3,814) = 579.61, *p* < 0.001 in sample 2. As shown in Table 4, the univariate tests for each cluster variable (in each sample) were significant and demonstrated that cluster membership explained more than 50% variance of the three cluster variables. Table 4 also displays per sample whether the four clusters differed in each level of study motives. Except for cluster 1 and 4 (equal extrinsic motives) and cluster 2 and 4 (equal self-transcendent motives), the composition of each cluster was significantly different from the others. Following earlier research (Breckenridge, 2000; Vansteenkiste et al., 2009), we empirically tested the stability of the cluster solution in each sample through a double-split cross-validation procedure. The first step of the procedure entailed randomly splitting the samples into halves (i.e., creating two subsamples per sample). We then performed *k*-means cluster analysis on each subsample whereby the clusters were subsequently compared for agreement with the original cluster using Cohen’s kappa (κ). An agreement of at least 0.60 is considered acceptable after the kappa’s of each subsample are averaged (Vansteenkiste et al., 2009). Average values of 0.94 in sample 1 and 0.60 in sample 2 granted evidence for the stability of our four-cluster solution.

**TABLE 3:** Indices for *k*-means profile solutions.

Number of profiles	Variance explained						Profile sizes	
	ST		SO		EX			
	Sample 1	Sample 2	Sample 1	Sample 2	Sample 1	Sample 2	Sample 1	Sample 2
3	0.40	0.41	0.42	0.42	0.55	0.56	188, 256, 319	218, 285, 312
4	0.53	0.53	0.57	0.55	0.53	0.52	132, 179, 210, 242	119, 210, 221, 265
5	0.57	0.59	0.59	0.61	0.60	0.60	93, 114, 172, 192, 192	77, 140, 162, 214, 222

*The abbreviations refer to the following: ST, self-transcendent motives; SO, self-oriented motives; EX, extrinsic motives.*

**TABLE 4:** Cluster centers and multivariate analysis of variance.

	Cluster 1:		Cluster 2:		Cluster 3:		Cluster 4:					
	High-impact mindset		Low-impact mindset		Social-impact mindset		Self-impact mindset					
									*F*		η^2^	
	Sample 1	Sample 2	Sample 1	Sample 2	Sample 1	Sample 2	Sample 1	Sample 2	Sample 1	Sample 2	Sample 1	Sample 2
*n*	210	265	132	119	242	210	179	221				
	(27.5%)	(32.5%)	(17.3%)	(14.6%)	(31.7%)	(25.8%)	(23.5%)	(27.1%)				
Gender (% male)	61.9%	59.5%	84.8%	78.2%	55%	59.5%	74.9%	74.5%				
Ethnicity (% non-western)	23.8%	15.1%	15.1%	11.8%	19%	16.2%	12.3%	11.3%				
Self-transcendent motives	1.05c	0.93c	−0.74a	−0.81a	0.04b	0.08b	−0.74a	−0.76a	284.76***	307.68***	0.53	0.53
Self-oriented motives	0.84d	0.75d	−1.44a	−1.58a	−0.12b	−0.18b	0.24c	0.06c	335.08***	331.43***	0.57	0.55
Extrinsic motives	0.64c	0.56c	0.13a	−0.14a	−1.04b	−1.15b	0.55c	0.50c	284.89***	289.13***	0.53	0.52

Letters denote post hoc comparisons based on Tukey’s HSD between clusters; different letters mean significant different cluster centers (NB. The letters do not refer to comparisons between samples). *p < 0.05, **p < 0.01, ***p < 0.001.

### Characteristics of the Clusters

Based on theoretical and statistical criteria, the four-cluster solution was chosen which revealed four meaningful profiles highlighting specific patterns of variables. The descriptions of these four profiles are unpacked below. We named each of the profiles and used the term “mindset” for each of the profiles, because we believed that this represents the frame of mind students have when they start their study. We also used the term “impact” for each profile, because impact refers in the current context to having an influence on someone or something through studying at university.

#### High-Impact Mindset (*n* = 210; 27.5% in Sample 1 and *n* = 265; 32.5% in Sample 2)

The first profile is characterized by a relatively high level of self-transcendent motives, a high level of self-oriented motives, and a high level of extrinsic motives. Hence, these students showed high levels of confidence about their reasons for studying. They reported to go to university both to improve themselves but also the world around them. As they aim to positively affect every aspect of their lives through their study program, we labeled this student profile the *high-impact mindset*. Interestingly, gender was distributed more equal in this profile than the overall sample (total sample = 67.2% male students in sample 1 and 67% in sample 2) with a proportion of 61.9% male students in sample 1 and 59.5% in sample 2. In addition, this profile was composed of larger proportions of non-Western ethnic minority students compared to the overall sample (total sample = 18.1% in sample 1 and 14.1% in sample 2) with 23.8% in sample 1 and 15.1% in sample 2.

#### Low-Impact Mindset (*n* = 132; 17.3% in Sample 1 and *n* = 119; 14.6% in Sample 2)

The second and smallest profile is characterized by relatively low levels of self-transcendent motives and self-oriented motives, and moderate levels of extrinsic motives. These students reported less defined reasons for studying. Besides something that was expected or earning money in the future, they were mostly uncertain why they go to university. As these students seemed uninterested to proactively influence their personal development and indifferent to help others through their studies, we labeled this profile the *low-impact mindset*. This profile consisted for the largest part of male students, whereby the 84.8% in sample 1 and 78.2% in sample 2 were higher than the average of males in the samples (i.e., 67.2 and 67%). Furthermore, 15.1% of this profile was composed of non-Western ethnic minority students in sample 1 and 11.8% in sample 2; both proportions were a bit lower than the overall averages of 18.1% in sample 1 and 14.1% in sample 2.

#### Social-Impact Mindset (*n* = 242; 31.7% in Sample 1 and *n* = 210; 25.8% in Sample 2)

The third student profile is characterized by relatively moderate levels of self-transcendent motives and self-oriented motives, and relatively low levels of extrinsic motives. These students reported other-oriented reasons for studying as their most prevalent aspiration. Hence, these students have adopted a view that their university education particularly enables them to improve the conditions in society. Because their motivation for learning is grounded in having a positive effect on people and communities (including their own development), we labeled this profile the *social-impact mindset*. The gender distribution showed to be more equal (55% of male students in sample 1 and 59.5% of male students in sample 2) than the overall sample. The non-Western ethnic minority students comprised 19% of this profile in sample 1 and 16.2% in sample 2; both were slightly higher than the overall sample proportion.

#### Self-Impact Mindset (*n* = 179; 23.5% in Sample 1 and *n* = 221; 27.1% in Sample 2)

The fourth profile is characterized by relatively high levels of extrinsic motives, moderate levels of self-oriented motives, and relatively low levels of self-transcendent motives. These students indicated that they go to university merely to improve their own personal situation. Rather than aiming to positively influence other people and society, these students reported to have chosen their university studies purely out of self-interest. Based on this drive to pursue self-focused aims, we labeled the last student profile the *self-impact mindset*. Remarkably, the proportions of male students in this profile (74.9% in sample 1 and 74.5% in sample 2) are substantially larger than the averages of the samples. Furthermore, this fourth profile was composed of 12.3% of non-Western ethnic minority students in sample 1 and 11.3% in sample 2; both numbers were lower than the average proportion of the overall sample.

### Mindset Classification Tool

At this stage, we have found meaningful motivational mindset profiles and also replicated them. Since we suspect that the motivational mindsets are related to study success and intervention effects, it would be highly valuable for practice to be able to classify students in one of the four mindsets based on their motives for studying. However, practitioners (e.g., study advisors) have limited access and knowledge to perform a complex cluster analysis. In addition, practitioners do not always have access to large groups of students as the ones in the current study and performing cluster analysis in small student groups will likely not yield the same set of mindset profiles. We have therefore decided to develop a *classification tool* with which individuals can classify students using a simple methodology. Such a tool is not only valuable for practice, but also for future research. In a cluster analysis, *all* students are assigned to the clusters. Consequently, some students are allocated to one of the clusters in which they actually do not fit well. As we want to test hypotheses in future studies in order to draw further conclusions about the mindsets, it is important that the students who are classified with a certain mindset have a good fit with that mindset. Hence, the tool is also focused at achieving a more realistic method of classification.

To ensure simplicity, we decided that we had to give every student a score *level* instead of a numeric value on each of the three study motives dimensions and subsequently assign students with a certain pattern to one of the mindsets. We based these levels on the score relative to the rest of the sample. That is, we computed frequency tables of the three study motives dimensions and determined what the score range was per dimension. For each study motives dimension, we divided the range of the scale as it was answered by the sample in *three* parts. Based on which part of the scale the student scored, we assigned a level (i.e., low or middle or high) to the dimension and checked which mindset belonged to that pattern of motives.

Since there were three dimensions of study motives and three possible levels for each of these dimensions, a total of 27 possibilities of patterns existed (see Table 5). We deliberately assigned every pattern of levels (i.e., 27 possibilities) to one of the four mindsets. For instance, we would assign the *high-high-high* pattern to the high-impact mindset and we assigned the *low-low-low* pattern to the low-impact mindset. We based our allotment on the cluster means and on the descriptions of the mindsets. After allocating each pattern to a mindset, three independent raters categorized each pattern into one of the four mindsets as well. Similarly, the raters based their categorization on the results of the clustering and the descriptions provided. In a reliability comparison we found Cohen’s kappa to range from 0.56 to 0.80 which defined a moderate to good strength of agreement (Altman, 1991). After the raters categorized the patterns, we assessed their reasoning and utilized this input to calibrate the classification tool. Based on our discussions, we decided to leave out four patterns because each of these patterns did not fit properly with one of the four mindsets.

**TABLE 5:** Mindset allocation.

Mindset	Pattern of levels
	(ST – SO – EX)
High-impact	High – high – high
High-impact	High – high – middle
High-impact	High – middle – high
Low-impact	Low – low – low
Low-impact	Low – low – middle
Low-impact	Low – low – high
Low-impact	Low – middle – low
Low-impact	Low – middle – middle
Low-impact	Middle – low – middle
Low-impact	Middle – low – low
Social-impact	Middle – middle – low
Social-impact	Middle – high – middle
Social-impact	Middle – high – low
Social-impact	High – high – low
Social-impact	High – middle – low
Social-impact	High – middle – middle
Social-impact	High – low – low
Self-impact	Low – middle – high
Self-impact	Low – high – middle
Self-impact	Low – high – high
Self-impact	Middle – middle – high
Self-impact	Middle – low – high
Self-impact	Middle – high – high
NC	Low – high – low
NC	Middle – middle – middle
NC	High – low – high
NC	High – low – middle

*The abbreviations refer to the following: ST, self-transcendent motives; SO, self-oriented motives; EX, extrinsic motives; and NC, non-classifiable.*

Finally, we applied the classification tool to sample 2 and the distribution over the four mindsets in sample 2 showed to be similar as in the original cluster analysis with 215 students in the high-impact mindset, 95 students in the low-impact mindset, 198 students in the social-impact mindset, 205 students in the self-impact mindset, and 102 (13.9%) students in the residual group. To further examine the tool, we executed a reliability comparison between the classification tool and the original cluster analysis. Cohen’s kappa showed to be 0.70 which indicated a good agreement (Altman, 1991). Moreover, we inspected the students in the residual group and noticed that 89 of the 102 students specifically belonged to one of the four patterns (i.e., middle–middle–middle). Interestingly, these students were proportionally allocated in the cluster analysis to mindset 1, mindset 3, and mindset 4 as if they were randomly assigned. This substantiated our decision for a residual group and minimize the noise in the classification.

## Discussion

### Student Profiles

**FIGURE 2:**
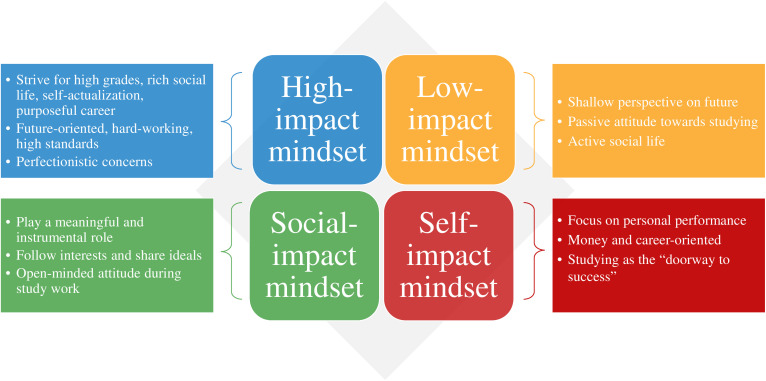
Characterizations of the four motivational mindsets.

The goal of the present study was to examine the interplay between three dimensions of study motives and to explore whether a set of meaningful motivational mindset profiles could exist among first-year students. The central research question was: “Can meaningful motivational mindset profiles, combining the three dimensions of study motives, be identified among first-year university students?” We discovered four distinct, meaningful, and useful motivational mindset profiles of students when combining three dimensions of study motives in our first sample of first-year students. Subsequently, we found a similar set of profiles when we replicated the exploration in a second sample of first-year students. We aimed to capture the multidimensionality of study motives by labeling them as *motivational mindsets* and, as this study concentrates on the conceptualization of the profiles, we like to put forward an illustration of each of these four mindsets. In addition, the illustrations are summarized and displayed in Figure 2.

Reports of the students in the *high-impact mindset* profile suggest that they strive for high grades, a rich social life, self-actualization, and a purposeful career. These students wish to thrive on every aspect of their lives. Consequently, due to the many tasks on their plate, they may experience high levels of engagement and fatigue simultaneously during the academic year (Salmela-Aro and Read, 2017). Feelings of drive but also pressure may be generated inwardly; yet, outside pressure such as from parents and from peers could be influential as well. Based on their scores, the high-impact mindset group members could also be prone to giving socially desirable answers. Moreover, the students with this mindset seem to have the tendency to work hard; they are hungry for challenges, future-oriented, and maintain high standards. Hence, both perfectionistic strivings and perfectionistic concerns can be characteristics that include the attitude of these students (Stoeber and Otto, 2006).

On the contrary, students in the *low-impact mindset* profile seem to possess a shallow perspective on why they go to university. Their scores suggest that they do not learn to develop themselves nor to have a positive influence on something bigger than themselves. This mindset likely includes a large number of students that attend university because it is expected of them. We could predict that students with a low-impact mindset might aim to pass the first year of university if possible with the minimum required grades. A significant part of these students might not even be sure why they have chosen this particular study program. Since these students indicate that they merely go to university for reasons such as having fun and making friends, many students with the low-impact mindset presumably hold a passive attitude toward their study work and will only learn at moments when they absolutely need to.

The students in the *social-impact mindset* profile pertain to a focus on self-growth and making the world a better place. These students learn to expand their knowledge and skills to play a meaningful and instrumental role. Students with a social-impact mindset probably desire to obtain their university degree in a fruitful manner. They view their studies as a means to personal development and they also gain fulfillment from learning which ultimately contributes to a higher purpose. These students tend to follow their interests and share their ideals and they are likely to possess a critical yet open-minded attitude during study work (Kosek, 1995).

Finally, the students in the *self-impact mindset* profile showed to be mostly self-oriented, which can translate to self-centeredness and this might possibly permeate in a sharp and perhaps even argumentative attitude during study work. These students may view their study program as a status symbol and, as such, they focus strongly on their personal performance. They could have the tendency to draw self-worth from their studies and regard their study as the ‘doorway to success.’ Media, parents, peers, and other role models can have a large influence on students with this mindset. The primary career-orientation of the students with a self-impact mindset seems to obtain a good and well-paid job. These students with their main focus on extrinsic rewards may well aim to realize this ambition by starting their own business and becoming an entrepreneur (Lechner et al., 2018).

### Strengths, Limitations, and Future Research

There are certain limitations worth discussing. First, because we used self-report assessments to measure study motives, students could have responded in a socially acceptable manner instead of a true reflection of their motives for studying. Hence, a limitation entails a potential method bias due to social desirability (Podsakoff et al., 2003). Future studies should consider this and could, for example, include a social desirability scale to determine how many respondents have the tendency to give socially desirable answers and possibly exclude participants from the research. Another limitation could be the study motives measurement instrument; specifically, the extrinsic motives subscale did not show high assessment quality. To elucidate this matter, we investigated the measurement model of the scale. In a measurement model, the relationships are described between a construct and its measures (i.e., items). In our case, we suspect that we could be dealing with a formative measurement model (specifically Type I, for a related discussion on the types of formative models see Diamantopoulos et al., 2008) because the items form and define the extrinsic motives dimension of our multidimensional construct. If the extrinsic motives subscale is formative (which we cannot test), it implies that the quality checks of this dimension of the instrument are unusable which could explain the inadequate assessment outcomes (Diamantopoulos et al., 2008). Additionally, limited procedures regarding the adaptation and translation of the motives for studying scale were followed. Although this does not depreciate the value of the results, future studies could apply recommended methods more thoroughly (e.g., Geisinger, 1994; Van Widenfelt et al., 2005). Furthermore, in the data we have, we could not assess the development of the profiles over time. As we assessed the motives at the start of the academic year, the question is whether students’ mindsets are stable over time. Prior research has indicated that this may not be the case and that the kind of study affects how mindsets of students change over time (e.g., Frank et al., 1993; Van Lange et al., 2011). Prior research also suggests that reflecting and writing about goals change the way students approach their studies and study environment (Schippers and Ziegler, 2019). Future research should investigate whether the mindsets remain stable longitudinally, and if not, whether they change in a predictable way. Lastly, since we collected data from students from a business university study program, our results are limited in that they may not be generalizable to students particularly dissimilar to these samples. Therefore, future studies could examine whether these same profiles emerge in other study programs and different age groups.

Despite several limitations, we particularly want to highlight the strengths of the current study. This study extends on the complementary value of person-centered research in education next to the traditional variable-centered approach. As students have multiple motives, pursuing a student-centered approach provided a rich and presumably more realistic representation of motivation in the first year at university. To our knowledge, this is the first study that combined these dimensions of motives for studying to construct student profiles and we demonstrated a new multidimensional perspective of student motivation. We validated the four profiles not only within each sample, but we also found almost identical profiles between the samples. In addition, this was the first study that investigated these dimensions of motives in a university setting. Notably, the two student samples were large and the sample characteristics were of similar composition.

After we identified four meaningful and statistically valid groups, we further enhanced their meaning by shaping them into mindsets. As the framework was tested rigorously, we alleged to their usefulness and decided to develop a mindset classification tool. Rather than needing to perform a complex cluster analysis or demand large student samples, this tool enables individuals to consistently classify students of any group size and in any setting into one of the motivational mindsets. First, the classification tool will primarily be utilized for further research. In these studies, we will concentrate on how the motivational mindsets relate to key academic outcomes. We will test hypotheses whether academic success and student wellbeing can be predicted based upon these mindsets. In addition, we might be able to explain the effectiveness of educational interventions. Students with a certain mindset can potentially experience less benefit from an intervention compared to students with another mindset. We need further insight into the differences between students’ motivational mindset for theoretical purposes and, especially, to further understand and tailor interventions that aim to enhance academic performance and wellbeing. The first year of university is a critical time for students. Hence, it is essential to know what mindsets toward studying exist when students enter university, if these mindsets affect academic functioning, and how they can be impacted positively.

## Conclusion

With our study we wanted to take a new step in mapping out the differences in motivation of students in higher education. Overall, this exploratory study lends support to the existence of four different and meaningful motivational mindsets toward studying among first-year university students. In addition to validating a new paradigm, we developed a mindset classification tool to further use these mindsets and conduct more imperative research on positive youth development in education.

## Data Availability Statement

The raw data supporting the conclusions of this article will be made available by the authors, without undue reservation to any qualified researcher.

## Ethics Statement

The studies involving human participants were reviewed and approved by Internal Review Board of the Erasmus Research Institute of Management, Erasmus University Rotterdam. Written informed consent for participation was not required for this study in accordance with the national legislation and the institutional requirements.

## Author Contributions

JH concepted and designed the study, performed the statistical analysis, and has written the draft of the manuscript. AS assisted in performing the statistical analysis. AS, MS, and GS contributed to the conception and design of the study. All authors contributed to revise, read, and approved the submitted version of the manuscript.

## Conflict of Interest

The authors declare that the research was conducted in the absence of any commercial or financial relationships that could be construed as a potential conflict of interest.
